# Defining the Scope of Digital Public Health and Its Implications for Policy, Practice, and Research: Protocol for a Scoping Review

**DOI:** 10.2196/27686

**Published:** 2021-06-30

**Authors:** Ihoghosa Iyamu, Oralia Gómez-Ramírez, Alice X T Xu, Hsiu-Ju Chang, Devon Haag, Sarah Watt, Mark Gilbert

**Affiliations:** 1 School of Population and Public Health University of British Columbia Vancouver, BC Canada; 2 British Columbia Centre for Disease Control Vancouver, BC Canada; 3 Canadian Institutes of Health Research (CIHR) Canadian HIV Trials Network Vancouver, BC Canada

**Keywords:** digital health, public health, prevention, scoping review, protocol

## Abstract

**Background:**

There has been rapid development and application of digital technologies in public health domains, which are considered to have the potential to transform public health. However, this growing interest in digital technologies in public health has not been accompanied by a clarity of scope to guide policy, practice, and research in this rapidly emergent field.

**Objective:**

This scoping review seeks to determine the scope of digital health as described by public health researchers and practitioners and to consolidate a conceptual framework of digital public health.

**Methods:**

The review follows Arksey and O’Malley’s framework for conducting scoping reviews with improvements as suggested by Levac et al. The search strategy will be applied to Embase, Medline, and Google Scholar. A grey literature search will be conducted on intergovernmental agency websites and country-specific websites. Titles and abstracts will be reviewed by independent reviewers, while full-text reviews will be conducted by 2 reviewers to determine eligibility based on prespecified inclusion and exclusion criteria. The data will be coded in an iterative approach using the best-fit framework analysis methodology.

**Results:**

This research project received funding from the British Columbia Centre for Disease Control Foundation for Population and Public Health on January 1, 2020. The initial search was conducted on June 1, 2020 and returned 6953 articles in total. After deduplication, 4523 abstracts were reviewed, and 227 articles have been included in the review. Ethical approval is not required for this review as it uses publicly available data.

**Conclusions:**

We anticipate that the findings of the scoping review will contribute relevant evidence to health policy makers and public health practitioners involved in planning, funding, and delivering health services that leverage digital technologies. Results of the review will be strategically disseminated through publications in scientific journals, conferences, and engagement with relevant stakeholders.

**International Registered Report Identifier (IRRID):**

DERR1-10.2196/27686

## Introduction

Over the past 2 decades, there has been rapid development and proliferation of digital technologies and their concomitant application to achieve health objectives [1,2]. Their applications offer great potential to transform the speed, efficiency, capacity, and impact of health services and programs, including in public health [3-5]. In May 2018, the World Health Organization Member States acknowledged this reality by unanimously approving the World Health Assembly Resolution on Digital Health [1]. This resolution recognized the potential value of digital technologies in achieving universal health coverage and the health-related aims of the United Nations Sustainable Development Goals [1].

Many public health initiatives and systems have integrated digital technologies in their operations. For instance, the Global Public Health Intelligence Network, maintained by the Public Health Agency of Canada, has leveraged big data capacity for global infectious disease surveillance [6]. Health prevention and promotion programs have leveraged social media strategies with the aim of expanding their reach [5]. Health services across the continuum of care, from clinical services to community prevention services, have adopted digital technologies. In 2019, the United Kingdom’s National Health Service launched a digital-first primary health strategy to increase health care coverage by securing online access to health records for all patients by April 2020 and access to virtual consultations for all patients by 2021 [7].

The term “digital public health” was first mentioned in Public Health England’s digital strategy in 2017 [8]. This term was used to refer to a re-imagination of public health that blends established public health wisdoms with new digital concepts and tools. However, digital public health is not a field well conceptualized and is characterized by ambiguity and confusing terminologies in the literature [9-11]. For example, digitization, digitalization, and digital transformation of public health services have often been used in varied contexts to refer to digital public health. However, these terms refer to distinct processes in many fields [11]. Further, despite the high proliferation of publications related to digital public health, especially in light of COVID-19, consensus on the definition, based on evidence, is yet to be achieved [1,4]. More specifically, the scope of digital public health needs to be clearly defined, including the technologies applied and the potential benefits, harms, and unintended or negative consequences of adopting digital technologies in population and public health. Similarly, the human resource and systems capacities required to fully take advantage of digital technologies in population and public health remain to be laid out [1,2].

Rather, enthusiasm for the application of digital technologies in public health has stimulated the proliferation of multiple, but often fleeting, digital health interventions. This proliferation increasingly diversifies the field, but very few interventions have been adopted at a large scale (eg, at a state or national level) where their potential benefits can become reality [1,12]. Complex issues regarding the impact of digital health interventions on reducing or widening health equity disparities and their ethical implications remain unresolved [13]. Evidence on these issues is crucial to thoughtfully implement and evaluate digital technologies and prevent unnecessary diversions of support and funding from other well-established, nondigital interventions.

As a first step, conceptualizing the scope of “digital public health” is necessary to clearly understand the field and to create appropriate policies and operational environments required to ensure the realization of its potentials. The European Public Health Association (EUPHA) has taken great strides to address this issue, recently creating a framework that conceptually defines digital technologies, their features, and the potential benefits of digitalization in public health [10]. However, the framework does not specifically describe how digital technologies may be deployed in specific public health domains; how we may consider foundational principles of public health, including health equity and social justice in this process [14]; or how to identify relevant challenges in digital public health and potential solutions. It may also benefit from additional domains and benefits of digitalization in public health as this field rapidly evolves. While other conceptual frameworks on digital health exist from a health systems perspective and the application of digital technologies restricted to specific public health domains [15], to the best of our knowledge, none other than the EUPHA framework broadly conceptualize a consolidated approach to digital technologies across the domains of public health.

Through this scoping review, we aim to conceptualize “digital public health” and to expand on the EUPHA’s conceptual framework by examining how the scope of digital health is described by public health researchers and practitioners within published and grey literature. We also seek to outline relationships between digital technologies and the public health domains, identifying the benefits, challenges, and potential solutions to these challenges within the domains of digital public health.

## Methods

### Project Design

This scoping review will utilize the framework proposed by Arksey and O’Malley [16] for scoping reviews, while integrating improvements to the framework as suggested by Levac et al [17]. Scoping reviews have been suggested as a useful method for clarifying complex concepts [17]. The framework recommends organizing the scoping review into 5 compulsory stages and an optional sixth stage.

#### Stage 1: Identifying the Research Question

A preliminary review of literature in the field suggests that the conceptualization of “digital public health” is relatively recent [10,18-20]. Therefore, this scoping review will be more broadly focused on how “digital health” and closely related domains (eg, virtual health, mobile health [mHealth], eHealth) have been conceptualized and characterized within public health research and practice discussions. Our main research question is therefore: “How is digital health described, understood, and applied by public health researchers and practitioners within the context of public health?”

We anticipate that selected literature may include: conceptual descriptions of digital health in relation to public health practice, goals or purpose of applying digital technologies in public health, types of digital technologies applied in public health, amenability of various public health domains to digital health approaches, and potential benefits and challenges of digital technologies in public health.

#### Stage 2: Identifying Relevant Literature

To comprehensively identify literature relevant in conceptually defining “digital public health,” we will employ a broader analytical lens and search strategy that will enable us to capture the main defining issues, developments, and debates that shape this rapidly evolving field.

We recognize that public health and clinical medicine are distinct practices with significant areas of overlap. Therefore, we will restrict our scoping review to the application of digital health in public and population health, with accommodations for areas of overlap including disease prevention and control, quality of care, ethics, research, guidelines, decision support, training, and management [21]. However, facets of digital health as applied exclusively within clinical medicine will not be assessed in the review.

We will adopt the following working definitions of “digital health” and “public health” to guide the search strategy.

The World Health Organization defines digital health as “the field of knowledge and practice associated with the development and use of digital technologies to improve health. This is a broad umbrella term encompassing eHealth (including mHealth) and emerging areas such as the use of advanced computing sciences in big data, genomics, and artificial intelligence” [3].

**Figure 1:**
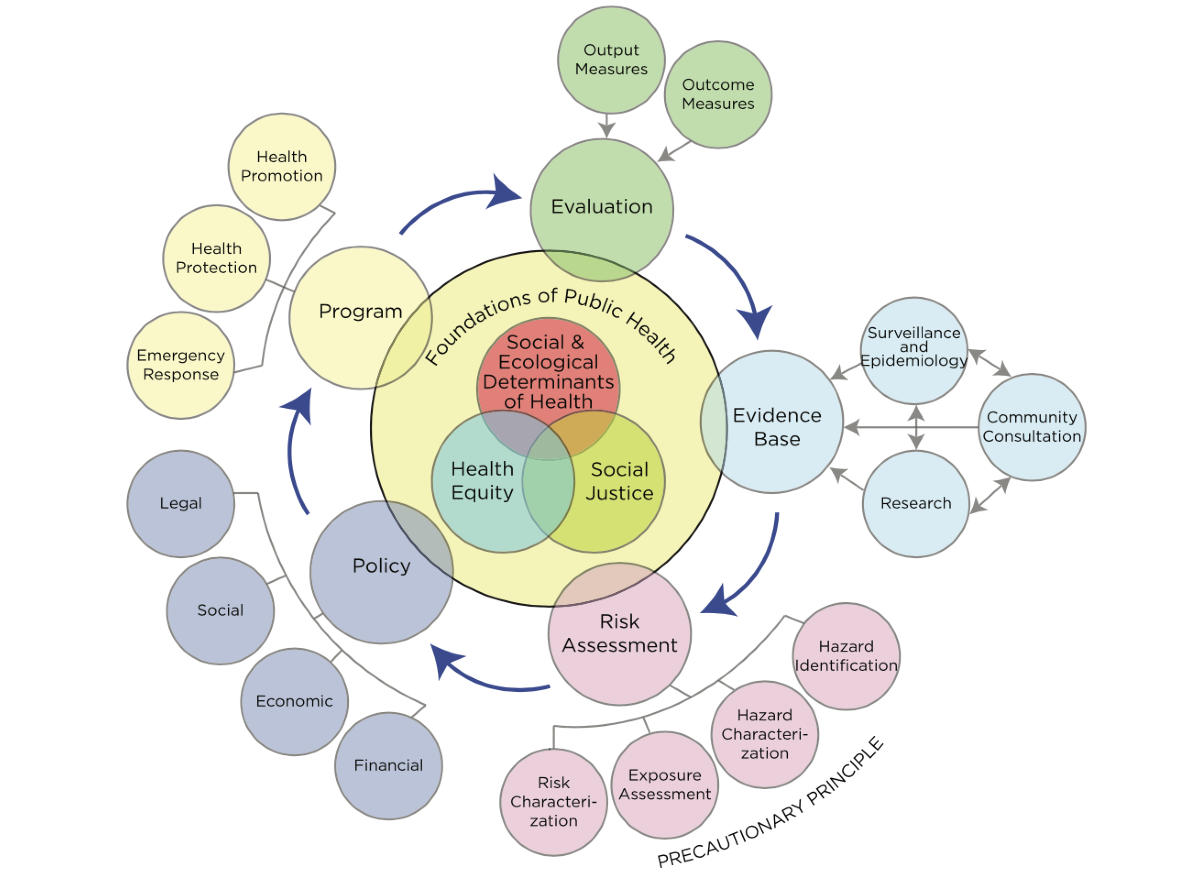
Canadian Public Health Association framework for public health.

We will apply the Canadian Public Health Association’s (CPHA) definition of public health as “an approach to maintaining and improving the health of populations that is based on the principles of social justice, attention to human rights and equity, evidence-informed policy and practice, and addressing the underlying determinants of health. Such an approach places health promotion, health protection, population health surveillance, and the prevention of death, disease, injury and disability as the central tenets of all related initiatives” [14]. This definition is illustrated in the CPHA’s framework (Figure 1).

Table 1 describes the inclusion and exclusion criteria that will be adopted for this scoping review. Publications that conceptually describe digital technologies or identify goals, benefits, challenges, and components of digital health from a public health or population health perspective will be included in this study. We will focus on the time period between January 2000 and June 2020 because our preliminary searches reveal that discussions about innovative health care services delivery in the internet age have proliferated within this timeframe.

We will search MEDLINE (Ovid) and Embase (Ovid) as bibliographic and citation databases for relevant literature on digital health. Grey literature searches will be conducted using Google Scholar and other agency or country websites as shown in Table 2.

**Table 1:** Eligibility criteria.

Parameter	Inclusion criteria	Exclusion criteria
Phenomenon of interest	Publications that broadly conceptualize and/or analyze digital health from a public health perspective	Publications evaluating or describing specific digital health programs or interventions
Health context	Publications that focus on health issues at the population level or population health outcomes, with a focus on preventive, community medicine, or public health (eg, environmental health, obesity, diabetes, stigma, antibiotic resistance, prevention of sexually transmitted and blood-borne infections)	Publications solely focused on the application of digital health in clinical contexts
Language	English	Not in English
Publication status	Published and grey literature	No full text, only abstract or short summary <500 words published
Year of publication	January 2000 and June 2020	None

**Table 2:** Agency and country websites searched for grey literature.

Country or jurisdiction	Agency
Intergovernmental	World Health Organization
Europe	European Public Health Association
Australia	Public Health Association of Australia, Government of Canada
Canada	Government of Canada, Public Health Agency of Canada, Canadian Institutes of Health Research, Canadian Public Health Association, National Collaborating Centres for Public Health, National Collaborating Centres for Determinants of Health, Canadian Agency for Drugs and Technologies in Health
United States of America	US Centers for Disease Control and Prevention, American Public Health Association
United Kingdom	UK Public Health Association, National Health Service

This strategy will aim to identify the intersection between terms related to “digital health” and “public health.” In consultation with a UBC librarian, we will determine a combination of keywords, Medical Subject Heading (MeSH) terms, and filters to maximize the comprehensiveness of our search. Preliminary keywords that have been proposed to be searched in title, abstract, and keyword database fields include the following for digital health: digital public health, digital health, mHealth, virtual health*, mobile health, e-health/ehealth, online health, internet-based health, web-based health/web*, computer-based health, digitalization/digit*, electronic health, health informatics, digital tools, digital technologies, telehealth/telemedicine, health informatics, social media, predictive algorithms, or connected care devices, artificial intelligence, machine learning methods, big data. Similarly, the following preliminary keywords have been proposed for public health: public health, health promotion, health prevention, health protection, health policy, health determinants, surveillance, health evaluation, public health ethics, health economics, risk assessment, epidemiology, community health, emergency preparedness, emergency response, health equity, social justice, social determinants.

The final search strategy (Multimedia Appendix 1) will be informed by a pilot search of Ovid MEDLINE and Ovid Embase. The intersection between digital health and public health will be identified using Boolean terms such as “and” to identify literature relevant to digital public health. Using the finalized search terms and strategy, returns will then be retrieved from each database.

Grey literature searches will be conducted using simpler search terms: “digital” and “public health.” This search will be conducted on Google Scholar. We will review the first 100 returns to identify relevant literature. Further, we will apply the same search strategy on government-specific websites and intergovernmental agency websites. To standardize searches across country and agency websites, we will parse our search through Google to each website. For example, to search the Government of Canada website [22], we will apply the search terms: “digital “public health” site:canada.ca” on Google to identify relevant publications from the website. We will review the first 100 returns for each search for grey literature. All retrieved publications will be exported to Covidence for citation management and review.

#### Stage 3: Study Selection

Study selection will be conducted in 2 phases. In phase 1, titles and abstracts of all literature retrieved from the searches will be reviewed to determine eligibility for full-text review based on the inclusion and exclusion criteria. To ensure reliability of the review process, 25% of titles and abstracts will be independently assessed by 2 members of the research team (II and AX), who will meet to discuss discrepancies and establish a consensus for the study selection protocols.

In phase 2, 2 members of the research team will independently review all full texts of publications and gray literature included from phase 1 using a structured framework (Multimedia Appendix 2). Reviewers will have access to the list of full texts selected by other members of the team where discrepancies will be appropriately discussed to establish consensus. A comprehensive list of literature reviewed, included, and excluded will be compiled and used to create a flowchart that describes the literature selection process.

#### Stage 4: Charting the Data

Our methodological approach will be based on the best-fit framework analysis as described by Carroll et al [23]. This method offers a pragmatic way of conducting qualitative evidence synthesis that builds on existing theoretic or conceptual frameworks to address “policy-urgent” questions. It involves prior selection of a conceptual model, reduction of the model to variables that form the a priori framework, and coding new data against the a priori matrix framework, while making additional themes where appropriate, to inform a resultant framework [23].

Our a priori framework will be informed by the EUPHA conceptual framework on digitalization in public health and the CPHA framework for public health. We will finalize an initial matrix codebook (Multimedia Appendix 3) to support data extraction. Definitions of each of the codes in the matrix will be discussed among the authors based on existing literature and agreed on before commencement of coding, which will be conducted using QSR NVIVO version 12. Data from the selected literature will be coded against themes of the a priori framework using an iterative approach [24]. Publication details will be recorded including article type, author(s), publication year, country, and continent. Themes included in the a priori framework as informed EUPHA and CPHA frameworks will include the digital technology discussed, features of digital health, foundations of public health considered, public health domains, and potential benefits of digital public health. We will also code challenges and recommendations. Where applicable, new themes will be added to the a priori framework based on inductive interpretation and constant comparison of new themes across reviewed publications.

Initial data familiarization and coding will be completed by 2 members of the research team. As recommended by Levac et al [17], 10% of the full texts will be initially coded into the a priori framework, and findings will be discussed to ensure consistency of the charting process and allow for refinement of the codebook as appropriate. Intersections between digital technologies and public health domains will be double coded to relevant codes. This will allow us to identify dominant technologies that have been applied in specific public health domains. Matrix coding summaries will be produced and used to visually map related findings to aid interpretation.

#### Stage 5: Collating, Summarizing, and Reporting the Results

Data organized in the charting process will be thematically analyzed. Heatmaps will be generated to identify the extent to which the literature has documented the application of digital health technologies in the various domains of public health and its public health benefits. This will further inform analyses.

The analyses will inform the consolidation of a working conceptual framework of digital public health that includes the public health foundations, domains of public health, digital technologies applied to the domains, features of the digital technologies, and potential benefits of digital technologies in each domain. Further, practice and research gaps, challenges, and potential recommendations to address these gaps will be identified in the literature.

Reporting will follow the Preferred Reporting Items for Systematic reviews and Meta-Analyses statement - Scoping Reviews (PRISMA-ScR) [25]. As advocated by Levac et al [17], data from the analysis will be presented as descriptive numerical summaries (frequency tables and charts) including article type, country and continent of the first author, year of publication, and public health domain discussed. The data will also be presented as narrative syntheses and conceptual maps as appropriate depending on the research question. Differences in perspectives across years of publication, country and regions, and fields of practice will also be presented as narratives and tables.

#### Stage 6: Consultation With Stakeholders

Finally, we will consult with population and public health stakeholders to validate our conceptual framework for digital public health and identified research and practice gaps. We will convene a workshop with public health stakeholders, including individuals with and without expertise in the application of digital technologies to public health (eg, public health nurses and physicians, social media and communications staff, health promotion leads, virtual health representatives, public health researchers). Using a World Café method, where small groups rotate through tables on different discussion topics, we will facilitate discussions about various aspects of the framework and will document feedback as required. These discussions will also inform a working understanding of policy implications tied to the framework. All of this information will be used to finalize our working conceptual model of digital public health and will inform ongoing collaboration to further explore this emergent field.

### Ethics and Dissemination

Ethical approval was not required for this scoping review as it is a synthesis from publicly available publications. Data generated from the review will, however, be stored in a secured network drive. Findings from the scoping review will be shared with relevant stakeholders including leaders of the CPHA and academia through targeted consultations as has been described, to consolidate a conceptual framework for digital public health and inform policy and practice. Findings will also be published as a final report and in peer-reviewed journals and scientific conferences. Finally, members of our research team will utilize output from this scoping review to directly facilitate the development of public health initiatives and strategies within British Columbia, Canada, and beyond.

## Results

**Figure 2:**
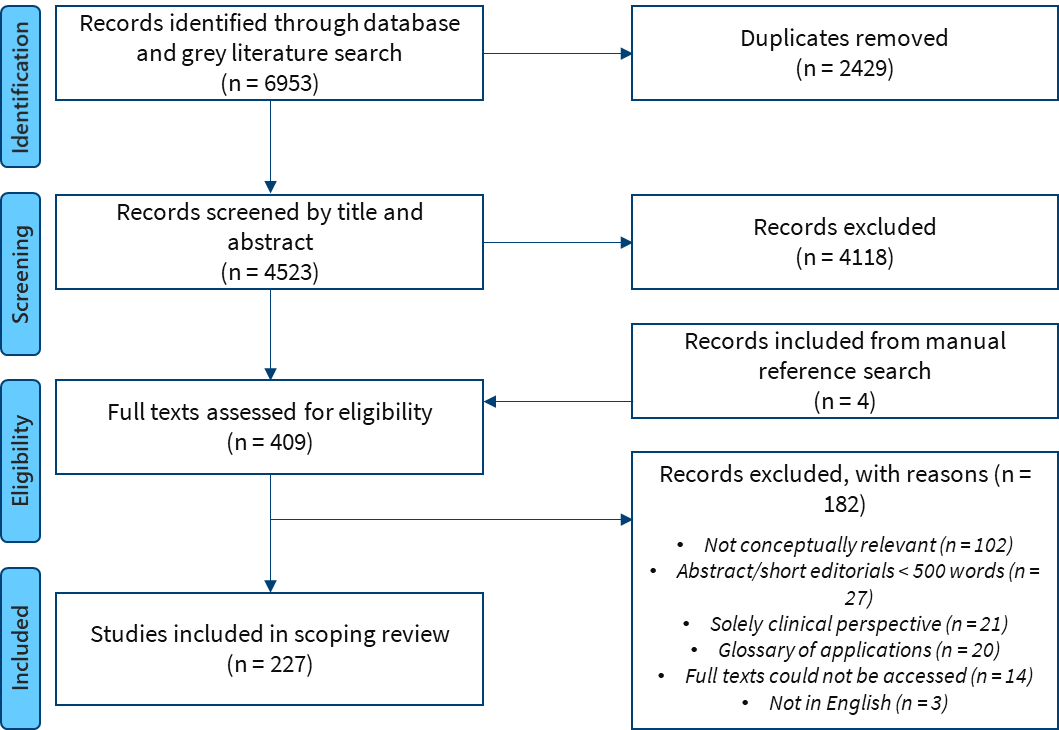
Preferred Reporting Items for Systematic Reviews and Meta-Analyses for Scoping Reviews (PRISMA-ScR) flow diagram of the search and study selection process.

This research project received funding from the British Columbia Centre for Disease Control (BCCDC) Foundation for Population and Public Health on January 1, 2020. The initial search was conducted on June 1, 2020 and returned 6953 articles in total (Figure 2). After deduplication, 4523 abstracts were reviewed, and 227 articles have been included in the review.

## Discussion

Digital public health continues to gain popularity as an emergent field of practice, especially in the context of COVID-19 [26]. As multiple public health initiatives integrate digital technologies to advance their objectives, effort is required to understand the scope of field, prevailing challenges, and potential solutions if public health researchers and practitioners are to appropriately support its ongoing development.

We envisage that the findings of the scoping review will contribute relevant evidence to health policy makers and public health practitioners involved in planning, funding, and delivering health services that leverage digital technologies. Given the broad scope of the review, we anticipate that we can potentially highlight already known fields and emergent and promising public health functions that can benefit from digital technologies, especially at scale.

This scoping review will focus on literature published between January 2000 and June 2020, excluding more recent articles. We acknowledge the recent upsurge in articles describing digital technologies in relation to public health emergency preparedness and response in the context of the COVID-19 pandemic. However, we consider it more relevant to focus on a more balanced view of digital public health that is not skewed by the discourse on the pandemic. Finally, given that our review strategy utilizes a nonspecific collection of publications, we have not undertaken a quality assessment of included articles in line with the framework for this scoping review [16].

## Appendix

Multimedia Appendix 1 Search strategy.

Multimedia Appendix 2 Full paper review framework.

Multimedia Appendix 3 Initial codebook.

## Abbreviations

BCCDC: British Columbia Centre for Disease Control
CPHA: Canadian Public Health Association
EUPHA: European Public Health Association
MeSH: Medical Subject Heading
mHealth: mobile health
PRISMA-ScR: Preferred Reporting Items for Systematic reviews and Meta-Analyses statement – scoping reviews
UBC: University of British Columbia
