# Supplementary material for: Defining the Scope of Digital Public Health and Its Implications for Policy, Practice, and Research: Protocol for a Scoping Review
Source: JMIR Res Protoc. 2021 Jun 30;10(6):e27686. doi: 10.2196/27686 (PMC8280811; doi:10.2196/27686)
Supplement: Multimedia Appendix 1 [file resprot_v10i6e27686_app1.docx]

**Appendix 1: Search strategy**

Database; Ovid MEDLINE(R) and Epub Ahead of Print, In-Process & Other Non-Indexed Citations, Daily and Versions(R) 2000 to June 1^st^, 2020

Search Conducted June 1^st^; all languages included.

| **Searches** |
| --- |
| 1. (mhealth or m-health).mp. [mp=title, abstract, original title, name of substance word, subject heading word, floating sub-heading word, keyword heading word, organism supplementary concept word, protocol supplementary concept word, rare disease supplementary concept word, unique identifier, synonyms] |
| 2. virtual health.mp. |
| 3. mobile health.mp. |
| 4. (ehealth or e-health).mp. [mp=title, abstract, original title, name of substance word, subject heading word, floating sub-heading word, keyword heading word, organism supplementary concept word, protocol supplementary concept word, rare disease supplementary concept word, unique identifier, synonyms] |
| 5. online health.mp. |
| 6. internet-based health.mp. |
| 7. computer-based health.mp. |
| 8. digit*.tw. |
| 9. electronic health.tw. |
| 10. health informatics.mp. |
| 11. web-based*.tw. |
| 12. telehealth.mp. |
| 13. Telemedicine/ |
| 14. Social Media/ |
| 15. predictive algorithms.mp. |
| 16. machine learning methods.mp. |
| 17. Machine Learning/ |
| 18. Big Data/ |
| 19. public health*.tw. |
| 20. Health Promotion/ |
| 21. health prevention.mp. |
| 22. health protection.mp. |
| 23. Health Policy/ |
| 24. health determinants.mp. |
| 25. Population Surveillance/ |
| 26. health evaluation.mp. |
| 27. health economics.mp. |
| 28. Risk Assessment/ |
| 29. Epidemiology/ |
| 30. community health.mp. |
| 31. emergency preparedness.mp. |
| 32. emergency response.mp. |
| 33. Health Equity/ |
| 34. Social Justice/ |
| 35. social determinants.mp. |
| 36. Artificial Intelligence/ |
| 37. digital public health.mp. |
| 38. digital health.mp. |
| 39. digitalization.mp. |
| 40. digital tools.mp. |
| 41. digital technologies.mp. |
| 42. web-based health.mp. |
| 43. 1 or 2 or 3 or 4 or 5 or 6 or 7 or 9 or 10 or 12 or 14 or 15 or 16 or 18 or 36 or 37 or 38 or 39 or 40 or 41 or 42 |
| 44. 19 or 20 or 21 or 22 or 23 or 24 or 25 or 26 or 27 or 28 or 29 or 30 or 31 or 32 or 33 or 34 or 35 |
| 45. 43 and 44 |
| 46. ((mhealth or m-health or virtual health or mobile health or (ehealth or e-health) or online health or internet-based health or computer-based health or electronic health or health informatics or telehealth or Social Media or predictive algorithms or machine learning methods or Big Data or Artificial Intelligence or digital public health or digital health or digitalization or digital tools or digital technologies or web-based health) and (public health* or Health Promotion or health prevention or health protection or Health Policy or health determinants or Population Surveillance or health evaluation or health economics or Risk Assessment or Epidemiology or community health or emergency preparedness or emergency response or Health Equity or Social Justice or social determinants)).tw. |
| 47. limit 46 to (english language and yr="2000 -Current") |
| 48. Public Health/ |
| 49. public health ethics.mp. |
| 50. 20 or 21 or 22 or 23 or 24 or 25 or 26 or 27 or 28 or 29 or 30 or 31 or 32 or 33 or 34 or 35 or 48 or 49 |
| 51. 43 and 50 |
| 52. ((mhealth or m-health or virtual health or mobile health or (ehealth or e-health) or online health or internet-based health or computer-based health or electronic health or health informatics or telehealth or Social Media or predictive algorithms or machine learning methods or Big Data or Artificial Intelligence or digital public health or digital health or digitalization or digital tools or digital technologies or web-based health) and (Health Promotion or health prevention or health protection or Health Policy or health determinants or Population Surveillance or health evaluation or health economics or Risk Assessment or Epidemiology or community health or emergency preparedness or emergency response or Health Equity or Social Justice or social determinants or Public Health or public health ethics)).tw. |
| 53. limit 52 to (english language and yr="2000 -Current") |
| 54. 1 or 2 or 3 or 4 or 5 or 6 or 7 or 10 or 12 or 14 or 15 or 16 or 18 or 36 or 37 or 38 or 39 or 40 or 41 or 42 |
| 55. 50 and 54 |
| 56. ((Health Promotion or health prevention or health protection or Health Policy or health determinants or Population Surveillance or health evaluation or health economics or Risk Assessment or Epidemiology or community health or emergency preparedness or emergency response or Health Equity or Social Justice or social determinants or Public Health or public health ethics) and (mhealth or m-health or virtual health or mobile health or (ehealth or e-health) or online health or internet-based health or computer-based health or health informatics or telehealth or Social Media or predictive algorithms or machine learning methods or Big Data or Artificial Intelligence or digital public health or digital health or digitalization or digital tools or digital technologies or web-based health)).tw. |
| 57. limit 56 to (english language and yr="2000 -Current") |
| 58. Public Health Surveillance/ |
| 59. 20 or 21 or 22 or 23 or 24 or 26 or 27 or 28 or 29 or 30 or 31 or 32 or 33 or 34 or 35 or 48 or 49 or 58 |
| 60. 54 and 59 |
| 61. ((mhealth or m-health or virtual health or mobile health or (ehealth or e-health) or online health or internet-based health or computer-based health or health informatics or telehealth or Social Media or predictive algorithms or machine learning methods or Big Data or Artificial Intelligence or digital public health or digital health or digitalization or digital tools or digital technologies or web-based health) and (Health Promotion or health prevention or health protection or Health Policy or health determinants or health evaluation or health economics or Risk Assessment or Epidemiology or community health or emergency preparedness or emergency response or Health Equity or Social Justice or social determinants or Public Health or public health ethics or Public Health Surveillance)).tw. |
| 62. limit 61 to (english language and yr="2000 -Current") |
| 63. 61 not "trial".mp. [mp=title, abstract, original title, name of substance word, subject heading word, floating sub-heading word, keyword heading word, organism supplementary concept word, protocol supplementary concept word, rare disease supplementary concept word, unique identifier, synonyms] |
| 64. 63 not "cross-sectional".mp. [mp=title, abstract, original title, name of substance word, subject heading word, floating sub-heading word, keyword heading word, organism supplementary concept word, protocol supplementary concept word, rare disease supplementary concept word, unique identifier, synonyms] |
| 65. limit 64 to (english language and yr="2000 -Current") |
