# Supplementary material for: Defining the Scope of Digital Public Health and Its Implications for Policy, Practice, and Research: Protocol for a Scoping Review
Source: JMIR Res Protoc. 2021 Jun 30;10(6):e27686. doi: 10.2196/27686 (PMC8280811; doi:10.2196/27686)
Supplement: Multimedia Appendix 2 [file resprot_v10i6e27686_app2.docx]

**Appendix 2: Framework for full text review**

1. Was access gained to the full paper of the citation
   - Yes, full paper accessed^1^
   - No, full text could not be downloaded^2^
   - Only abstract available or full article less than 500 words^3^

*If yes to 1, move to step 2, else stop the review and indicate reason for exclusion*

1. Is the full text in English?
   - Yes^3^
   - No^4^

*If option 3 (yes) selected, move to step to the next step, else stop the review and indicate reason for exclusion as “Language not English”*

1. Does the paper broadly conceptualize and/or analyze a digital health intervention/application from any public health perspective (as described in the CPHA framework)? ^[[1]](#endnote-1)^
   - Yes, it is a commentary/narrative review/systematic review broadly applying digital health in public health^5^
   - Yes, it presents a conceptual analysis of digital health in public health, but as a section of a broader public health or digital health discussion^6^
   - No, it is a primary study or presents a report of a specific digital health intervention^7^
   - No, it is a commentary focusing entirely on describing a specific digital health intervention^8^
   - No, it is a systematic review that presents a glossary of specific digital health interventions^9^
   - No, it discusses digital health **entirely** from a clinical perspective^10^
   - Can’t tell^11^

*If option 5 or 6 selected, include the paper, else exclude and indicate “not conceptual” for options 7-9, or “clinical/technical perspective” for option 10. If option 11 is selected, indicate “maybe” and discuss with co-reviewer.*

1. In some of the papers, the CPHA dimensions may not be explicitly stated. Using our own judgement and knowledge of public health will be useful in determining the relevance of the paper to this study. For example, a digital health intervention may be described in “disaster preparedness” which is not directly described in the framework, but it aligns with emergency response. This kind of paper can be included if the other inclusion criteria are met. [↑](#endnote-ref-1)
