# Supplementary material for: Defining the Scope of Digital Public Health and Its Implications for Policy, Practice, and Research: Protocol for a Scoping Review
Source: JMIR Res Protoc. 2021 Jun 30;10(6):e27686. doi: 10.2196/27686 (PMC8280811; doi:10.2196/27686)
Supplement: Multimedia Appendix 3 [file resprot_v10i6e27686_app3.docx]

**Appendix 3: Initial Matrix Codebook to Guide Best-Fit Framework Analysis**

| Building blocks - Public Health Domains |  |
| --- | --- |
| Evaluation | Assessment of a policy or program's delivery of agreed deliverables and the mediation of the outputs in achieving desired outcomes.^[1]^ |
| Community consultation | Processes that enable community members to be active contributors through collaboration and involvement to public health initiatives that seek to establish positive change within the community.^[1]^ |
| Research | Processes and activities that contribute to generalizable knowledge, inform public health practice and policy, and are targeted to develop, implement and evaluate efficient ways of health promotion, protection and disease prevention.^[1]^ |
| Surveillance and epidemiology | The continuous, systematic collection, analysis and interpretation of health-related data needed for planning, implementing and evaluating public health practice.^[1]^ |
| Policy | Defined as the principles or protocols adopted or proposed by a government, party, business or individual that provide a definitive course of action, and guide or determine present or future decisions.^[1]^ |
| Emergency preparedness | Activities that provide the capacity to respond to acute harmful events that range from natural disasters to infectious disease outbreaks and chemical spills. Includes prevention, preparedness, response and recovery activities.^[1]^ |
| Health promotion | A mix of activities that assist individuals and communities in taking charge of their personal health. Includes health education and related organizational, economic and political interventions designed to facilitate behavioral and environmental changes conducive to health.^[1]^ |
| Health Protection | Activities that address negative influences on health, and include diverse interventions such as testing of food and water supplies, surveillance to identify and track infectious disease outbreaks.^[1]^ |
| Risk assessment | Actions taken to estimate the nature and likelihood of negative health outcomes in the populations and includes occupational, environmental, social and behavioral risks.^[1]^ |
| **Foundations of Public Health - Considerations** |  |
| Health Equity | The absence of avoidable or remediable differences in health among groups of people, whether those groups are defined socially, economically, demographically or geographically.^[1]^ |
| Social and Ecological Determinants of Health | Conditions in which people are born, grow, live, work and age and how these factors intermingle to define the health situation specific to the population.^[1]^ |
| Social Justice | Measures that enable people to lead fulfilling lives and be active contributors to community. It includes measures to ensure that the whole population has equitable access to the public health initiative to minimize preventable deaths, injury and disability.^[1]^ |
| **Digital Health Technologies** |  |
| Artificial intelligence (including predictive analytics, speech recognition and natural language processing) | Describes the utilization of software, algorithms, including machine-learning algorithms, to emulate human cognition in the analysis, interpretation and comprehension of complicated healthcare data.^[2]^ |
| Big Data | Technologies and techniques to collect, link, and analyze massive volumes of data, including patient records, otherwise too large and complex for traditional technologies to assess.^[2]^ |
| Distributed ledger technologies (including blockchain technologies and cryptocurrency) | A consensus of replicated, shared and synchronized data that is geographically spread across multiple sites, countries or institutions. In healthcare, it may apply to health information sharing channels for personal health records.^[2]^ |
| Drones | Unmanned aerial vehicles (UAVs) which may be used to provide timely delivery of medical equipment and materials like lab samples, blood, and vaccines especially in emergencies.^[2]^ |
| Genomics | “Reading the genome” is the capacity to ‘read’ an individual’s genome and capture the variation within it. It represents a unique insight into the molecular characterization of diseases. Genomic analyses will involve all areas of healthcare and impact the patient’s entire journey, from diagnosis to monitoring and treatment. “Genome-editing”, genomic engineering strategies and synthetic biology tools holds the promise of benefiting patients through the ability not only to read but also to ‘write’ genomic information.^[2]^ |
| Internet of things | A blend of medical devices, and software that allow users generate, monitor, interpret and share health data across interoperable platforms to optimize health outcomes.^[2]^ |
| mHealth (smartphone applications) | Smartphone apps integrate education, symptom reporting and potential rehabilitation to improve self-management of diseases and order repeat prescriptions. They also facilitate self-monitoring illness progression and behavioral risk factors.^[2]^ |
| Robotics | Technologies which allow advanced functionality to patients with physical disability or can provide patient support.^[2]^ |
| Social media | Online channels (websites and applications) that allow end-users generate and share content in a community. In health, this may apply to channels for generate and monitor health and fitness related data within patient communities.^[2]^ |
| Telehealth (Telemedicine) | Telemedicine involves the provision of clinical care from a distance using telecommunication and information technology, including text, audio and video consultation, and is required to deliver the same standard of care as face-to-face consultations.^[2]^ |
| Virtual and augmented reality | Virtual and augmented reality is an immersive technology combining computer-generated visual, auditory and other sensory data with the physical world. They are both tools for the delivery of care and a platform for healthcare education.^[2]^ |
| Wearables and Sensors | Sensors and wearables allow self and remote monitoring and include: point-of-care and self-tests and self-monitoring, wearables and video cameras for patient monitoring.^[2]^ |
| **Features of Digital Health Technologies** |  |
| Automation | Automation is the use of control systems and information technologies to make processes operate automatically. In healthcare, this is automation applies to decision support systems and flagging adverse clinical events.^[2]^ |
| Data analytics (incl Big Data, data transfer and interoperability) | The science of integrating large volumes of data from multiple data sources and assessing the data to inform decision making.^[2]^ |
| Interaction | Interaction allows end-users (including patients) to generate, interpret and monitor their own health data, empowering them and their health perspectives.^[2]^ |
| Personalization and Precision | The capacity to provide targeted, effective and efficient public health interventions that have been adapted to the population.^[2]^ |
| Prediction | The ability to detect early or predict adverse events, infectious disease outbreaks or health emergencies, drawing from large volumes of existing data.^[2]^ |
| **Potential Public health Benefits and advantages** |  |
| Care closer to people | Improvements in access to care enabled by digital technologies to bring health services closer to people and populations otherwise under-served.^[2]^ |
| Less expensive care | Improvements in access to care by reducing financial barriers and cost of health services, as enabled by digital technologies. ^[2]^ |
| People-centered Care | Supporting the empowerment of people and patients by centering and contextualizing their needs at the forefront of public health services.^[2]^ |
| Safer, faster and more efficient services | Positive changes to healthcare to allow safer, faster, and more efficient delivery and management of public health services.^[2]^ |
| Shift from cure to prevention | Ways to self-manage health that can positively impact behavioral risk factors for primary prevention; techniques/technology to identify populations at increased risk on a genetic basis, and predict response to treatment for secondary prevention.^[2]^ |
| **Challenges** | Challenges reported in the planning, development, implementation and evaluation of outcomes related to digital technologies, from all stakeholders. |
| **Recommendations** | Suggested recommendations and solutions to address the reported challenges in the planning, development, implementation and evaluation of digital technologies. |

*Definitions integrated from the Topol report, Odone et al., conceptual framework for public health digitalization and the Canadian Public Health Association (CPHA) framework.*

**References**

1. The Canadian Public Health Association. Public Health: A conceptual framework [Internet]. Vol. 2. Ottawa; 2017. Available from: https://www.cpha.ca/sites/default/files/uploads/resources/cannabis/cpha_public_health_conceptual_framework_e.pdf

2. Odone A, Buttigieg S, Ricciardi W, Azzopardi-Muscat N, Staines A. Public health digitalization in Europe. Eur J Public Health [Internet]. 2019 Oct 1;29(Supplement_3):28–35. Available from: https://academic.oup.com/eurpub/article/29/Supplement_3/28/5628048
